# Proteomic signatures in cerebrospinal fluid and their clinical associations in patients with ME/CFS

**DOI:** 10.1038/s41598-026-46965-1

**Published:** 2026-04-03

**Authors:** Björn Bragée, Peng Li, Danielle Meadows, Anna Widgren, Per Sjögren, Per Hamid Ghatan, Bo C. Bertilson, Wenzhong Xiao, Jonas Bergquist

**Affiliations:** 1 ME Center, Bragée Clinics, Stockholm, Sweden; 2 Division of Family Medicine and Primary Care, Department of Neurobiology, Care Sciences and Society, Karolinska Institute, Stockholm, Sweden; 3 The Open Medicine Foundation Computational Research Center for Complex Chronic Diseases, Massachusetts General Hospital, Harvard Medical School, Boston, MA 02114 USA; 4 The Open Medicine Foundation, Agoura Hills, CA USA; 5 Analytical Chemistry and Neurochemistry, Department of Chemistry for Life Sciences, BMC, Uppsala University, Box 599, Uppsala, Sweden; 6 The Open Medicine Foundation ME/CFS Collaborative Research Centre at Uppsala University, Uppsala, Sweden

**Keywords:** Myalgic encephalomyelitis/chronic fatigue syndrome (ME/CFS), Cerebrospinal fluid proteomics, Postural orthostatic tachycardia syndrome (POTS), Disease severity biomarkers, Biomarkers, Diseases, Neurology, Neuroscience

## Abstract

This study evaluated the cerebrospinal fluid (CSF) proteomes from 31 patients diagnosed with myalgic encephalomyelitis/chronic fatigue syndrome (ME/CFS). We quantified 902 proteins, each expressed in at least eleven samples, and systematically categorized clinical factors relevant to ME/CFS symptoms—including autonomic dysfunction, neuroinflammation and metabolic disturbances. Differentially expressed protein and pathway analyses evaluated protein features associated with both postural orthostatic tachycardia syndrome (POTS) status and disease severity among the patients, while ratio-based analysis further explored associations with severity ratings. Data are available via ProteomeXchange with identifier PXD076216. Neutrophil degranulation and platelet activation were enriched in patients with POTS, and several pathways, such as the complement cascade, coagulation-related pathways and IGFBP‑mediated insulin-like growth factor transport, were enriched in severe cases. Ratio-based analysis identified four biologically interpretable severity-associated protein ratios related to cellular stress, extracellular remodelling and immune–neuronal interaction. Together, these findings provide insight into the biological processes associated with clinical heterogeneity in ME/CFS and generate hypotheses for future validation in larger independent cohorts.

**Supplementary Information:**

The online version contains supplementary material available at 10.1038/s41598-026-46965-1.

## Introduction

Myalgic encephalomyelitis/chronic fatigue syndrome (ME/CFS) is a poorly understood and debilitating condition, with a lifetime prevalence of more than 275 million people worldwide^1–4^. Its diagnosis is symptom-based, relying on identifying hallmark symptoms of the disease, including persistent fatigue, pain, sleep disturbances, cognitive issues, and post-exertional malaise^1,5^. While the symptom-based criteria used for diagnosis are comprehensive, they are also subjective. The lack of an objective diagnostic test frequently leads to functional or even psychiatric diagnoses, sometimes hindering clinical management of underlying pathophysiological processes^6^. There is a pressing need to better understand the molecular mechanisms of the condition to avoid misinterpretation and to improve diagnosis and treatment.

Cerebrospinal fluid (CSF) proteomics provides an invaluable resource for uncovering the molecular mechanisms underpinning diseases of the central nervous system^7–9^. Given the cognitive symptoms, dysautonomia, and neuroinflammation associated with ME/CFS, the disease pathology likely involves the central nervous system^10^. Therefore, proteomic studies on CSF can offer insights into pathophysiological processes and biomarkers associated with symptomatology of ME/CFS.

There are several published studies on the CSF of people with ME/CFS^11–16^. These studies provide evidence of central nervous system abnormalities in ME/CFS, specifically immune activation^12–14^ and metabolic differences^11^. They also highlight the presence of disease subgroups^12,14^ and overlap with diseases like fibromyalgia^16^ and post-treatment lyme disease^15^. While the studies contribute complementary information on the pathophysiology of ME/CFS—including disruption of lipid metabolism and linking metabolic abnormalities to immune dysfunction—there is no direct overlap in the molecules identified as significant.

This is the first study that examines in-depth the CSF proteomes of 31 ME/CFS patients in relation to disease severity and postural orthostatic tachycardia syndrome (POTS) status, using advanced high resolution mass spectrometry techniques. Importantly, protein ratios and pathway analysis are also reported. These analyses further elucidate the biological mechanisms underlying ME/CFS and provide potentially important protein signatures for future validation.

## Results

### Overview of proteomic and clinical data

Characteristics of participants (n = 31) are shown in Table 1. Participants were slightly obese, predominantly women (76%), and with poor subjective health (e.g., mean quality of life 34 ± 14 on a 0-to-100 scale). Routine blood chemistry analyses did not reveal any general deviations from normal, as reflected by cortisol and C-reactive protein (CRP) levels in Table 1.

**Table 1:** Characteristics of participants with ME/CFS who completed lumbar puncture and CSF collection (n = 31).

Characteristic	Value
Age in years, mean ± SD	45 ± 11
Female gender, n (%)	23 (74)
BMI kg/m^2^, mean ± SD	26.6 ± 5.6
Disease severity^A^, n	
Mild	3
Moderate	21
Severe	7
General hypermobility, n (%)	13 (42)
POTS prevalence, n (%)	9 (29)
Cortisol (nmol/L) n = 26, mean ± SD	403 ± 127
CRP (mg/L) n = 25, n (%)	
< 10	23 (92)
10–15	2 (8)
PROM derived symptomatology	
General pain (0–10), mean ± SD	4.7 ± 2.0
General fatigue (0–10), mean ± SD	6.4 ± 1.6
Headache prevalence, n (%)	27 (87)
Quality-of-life (0–100) n = 30, mean ± SD	35 ± 14

^A^according to Canadian Consensus Criteria and clinical consensus.

**Fig. 1:**
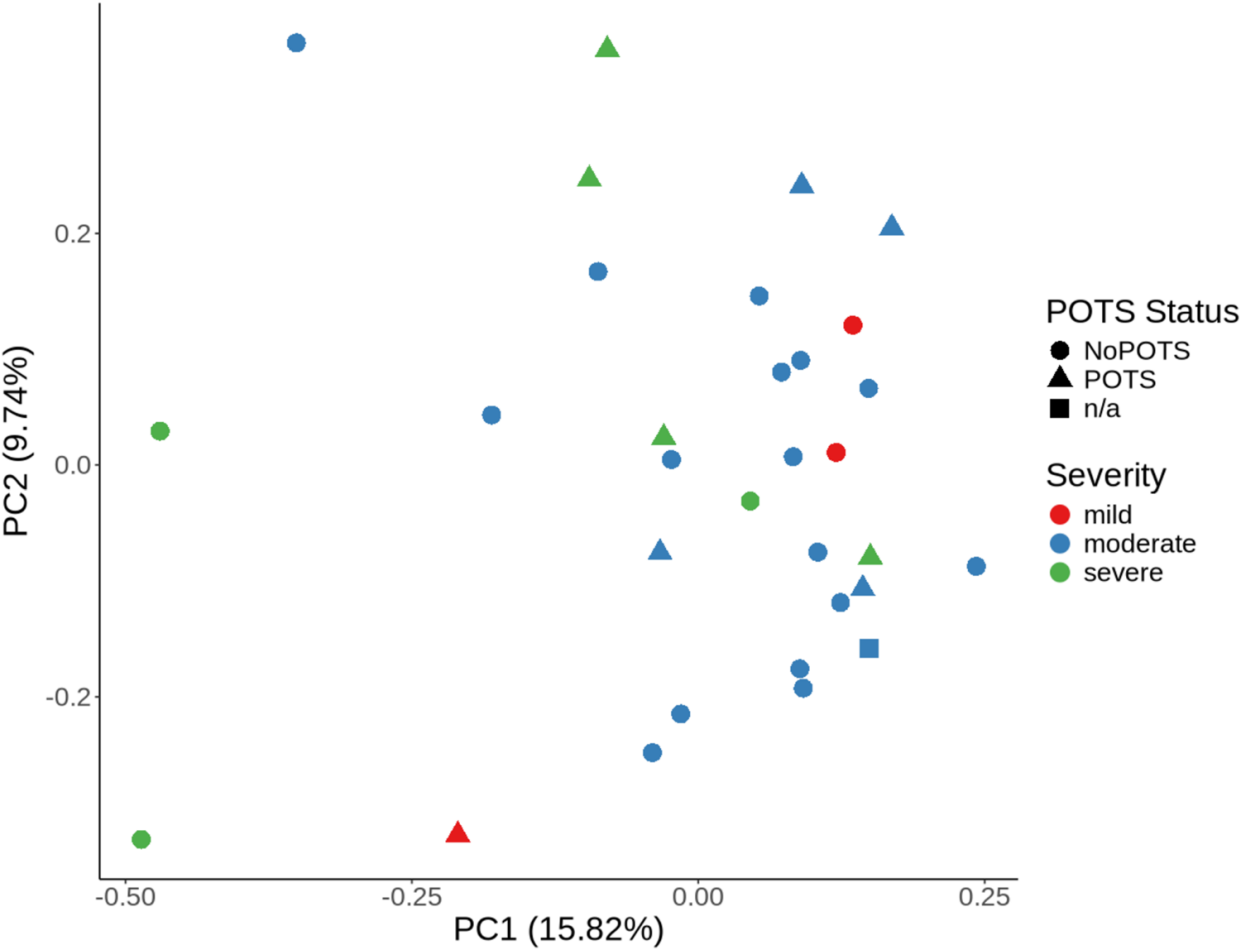
Principal component analysis of CSF proteomic profiles across ME/CFS severity and POTS status. Each point represents an individual CSF sample, colored by clinical severity and shaped by POTS classification.

A comprehensive analysis identified 902 proteins in CSF samples. On average, 782 proteins were quantifiable per sample. Proteomic variance was visualized through principal component analysis (Fig. 1).

### Proteomic alterations associated with POTS status in ME/CFS

**Fig. 2:**
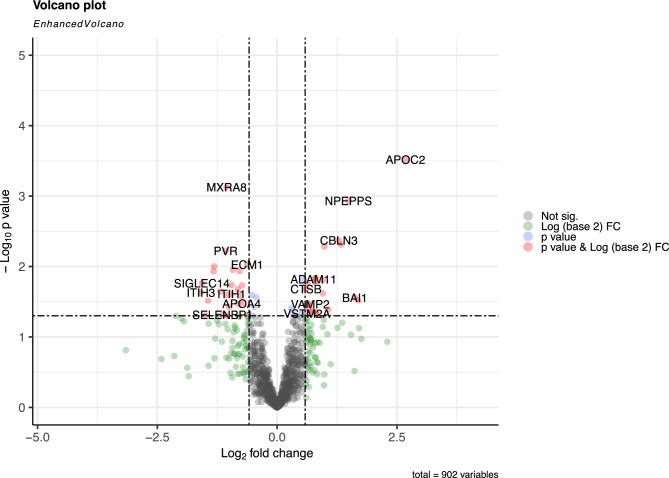
Volcano plot of differentially expressed proteins in ME/CFS patients with and without POTS (POTS n = 9 vs. NoPOTS n = 21). Each point represents a quantified protein, plotted by log₂ fold change (x-axis) and –log₁₀ p-value (y-axis). Proteins in the upper left and right quadrants show both large expression differences and strong statistical significance, indicating potential biological relevance.

Differentially expressed protein (DEP) analysis identified changes in protein levels between ME/CFS patients with and without POTS (Fig. 2). After adjustment for disease severity using a multivariable model, 47 proteins demonstrated nominal significance (p < 0.05, fold change > 1.5), including GAP43, MXRA8, and APOC2. Although no individual protein met the false discovery rate (FDR) threshold of < 0.05, Ingenuity Pathway Analysis (IPA)^17^ revealed coordinated biological signals (FDR < 0.05). Key canonical pathways enriched in the POTS comparison included Response to elevated platelet cytosolic Ca2 + , Neutrophil Degranulation, and Acute Phase Response Signaling. These findings suggest that the comorbid POTS phenotype in ME/CFS may be associated with innate immune activation and platelet-mediated inflammation.

### Proteomic alterations associated with ME/CFS disease severity

**Fig. 3:**
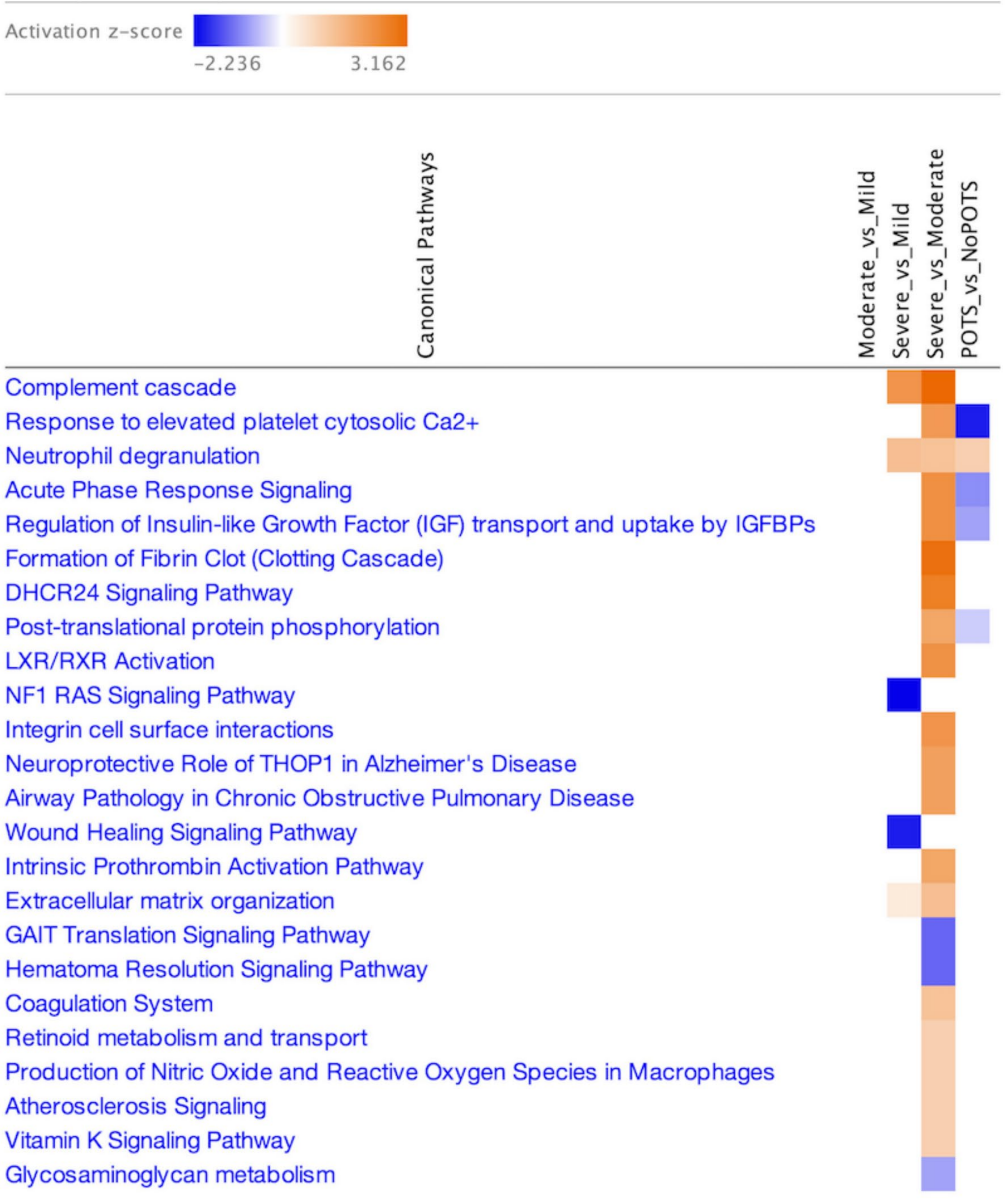
Enriched canonical pathways associated with ME/CFS clinical severity. Each cell represents a pathway’s predicted activation state (activation z-score) across severity group comparisons. Orange indicates pathway activation (positive z-score), while blue indicates inhibition (negative z-score), with color intensity reflecting the magnitude of the z-score.

To investigate biological mechanisms underlying symptom progression, we analyzed CSF proteomic profiles across clinically defined severity groups (mild, moderate, severe). A total of 141 unique proteins met nominal significance (p < 0.05, fold change > 1.5) across the three pairwise comparisons, with 102 proteins identified in Severe vs. Moderate, 50 in Severe vs. Mild, and 29 in Moderate vs. Mild. Pathway analysis applied to these severity-associated signatures highlighted several processes tracking with disease burden (FDR < 0.05). Notably, the Complement Cascade and Formation of Fibrin Clot pathways were specifically enriched in severe cases. This aligns with emerging hypotheses regarding thrombo-inflammation and complement dysregulation in severe neuro-immune conditions. Additionally, the enrichment of IGFBP-mediated insulin-like growth factor (IGF) transport suggests a role for metabolic or neurotrophic factor regulation in the pathophysiology of severe ME/CFS (Fig. 3).

### Severity-associated protein ratios reveal coordinated molecular shifts in ME/CFS

**Fig. 4:**
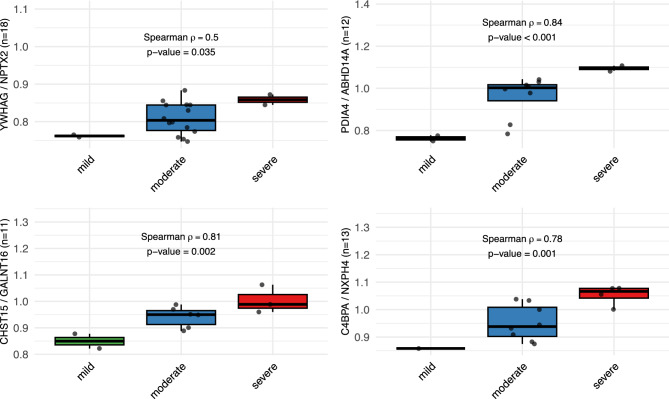
Protein abundance ratios correlated with ME/CFS clinical severity.

A recent study by Oh et al. (2025) identified the ratio of the levels of two proteins in the CSF—the ratio of YWHAG to NPTX2—as a marker of synaptic resilience and cognitive impairment in Alzheimer’s disease^18^. To assess its relevance in ME/CFS, this ratio was evaluated across the severity-defined subgroups. As shown in Fig. 4, the YWHAG/NPTX2 ratio increased with clinical severity, reflecting a similar trend to that seen in Alzheimer’s disease.

Expanding on this approach, a pairwise protein ratio analysis was applied using proteins differentially expressed across ME/CFS severity groups. To control for multiple hypothesis testing across all ratio comparisons, only protein ratios with FDR < 0.05 and absolute Spearman correlation (ρ) ≥ 0.75 were considered significant. Several ratios demonstrated significant associations with clinical severity after FDR correction (Table 2).

**Table 2:** Top 10 protein abundance ratios most strongly associated with ME/CFS clinical severity.

Rank	Pair	Sample size	Spearman		Kruskal–Wallis	
			ρ	p-value	p-value	q-value
1	**PDIA4_vs_ABHD14A**	12	0.836	0.0007	0.0214	0.043
2	TNFRSF21_vs_ABHD14A	13	0.815	0.0007	0.0185	0.043
3	RTN4RL2_vs_ABHD14A	13	0.815	0.0007	0.0185	0.043
4	**CHST15_vs_GALNT16**	11	0.814	0.0023	0.0346	0.044
5	DSC1_vs_ABHD14A	11	0.81	0.0025	0.0368	0.044
6	IL1RAP_vs_ABHD14A	11	0.81	0.0025	0.0368	0.044
7	CHST15_vs_CYTL1	13	0.8	0.0010	0.0209	0.043
8	**C4BPA_vs_NXPH4**	13	0.785	0.0015	0.0245	0.044
9	CHST15_vs_CD99	10	0.783	0.0074	0.049	0.046
10	CFP_vs_TYRO3	15	0.782	0.0006	0.0126	0.043

Three biologically interpretable and non-overlapping pairs were identified, meriting future consideration (also shown in Fig. 4): PDIA4/ABHD14A (ρ = 0.836), CHST15/GALNT16 (ρ = 0.814), C4BPA/NXPH4 (ρ = 0.785).

## Discussion

This study provides an in-depth characterization of the CSF proteome in ME/CFS, revealing exploratory signals linked to symptom severity and autonomic dysfunction. While the limited sample size constrained statistical power—particularly in subgroup and FDR-adjusted analyses—the integration of protein-level, ratio-based, and pathway analyses uncovered biologically plausible patterns across immune, metabolic, and neurofunctional domains.

### Complement activation, synaptic dysfunction, and extracellular matrix (ECM) remodeling in ME/CFS CSF

To minimize cross-platform bias, we verified our CSF findings at the pathway and result levels against external CSF datasets from similar disease cohorts.

Complement/coagulation. Our severity-linked enrichment of Complement cascade and Fibrin clot formation, together with a higher C4BPA/NXPH4 ratio, is consistent with disease-patterned complement signaling reported in CSF across neuroimmune conditions^15^ and with up-regulated complement (C9, CFD) and fibrinogen (FGA/FGB/FGG) in COVID-19 CSF^19^, supporting a reproducible neuroinflammatory axis.

Synaptic/neuronal processes. Our severity-associated YWHAG/NPTX2 ratio and nominal GAP43 signal align with independent CSF proteomics showing coordinated down-regulation of synaptic/neuronal proteins (e.g., NRCAM, NRXN2, SEMA7A, VGF) in COVID-19 versus controls, indicating synaptic stress is CSF-detectable in CNS-involved illness and consistent with current neuroimmune understanding^19^.

Extracellular matrix (ECM) remodeling / immune–neuronal coupling. Our Extracellular matrix organization pathway signal and CHST15/GALNT16 ratio (glycosaminoglycan sulfation / O-GalNAc transfer) support matrix remodeling; recent ME/CFS CSF immunophenotyping defined two immunotypes by MMP-1/-2/-10 with distinct cytokine milieus, reinforcing an ECM–inflammation axis relevant to clinical heterogeneity^14^.

### DEP, pathway, and ratio-based analyses

 DEP analysis of subgroups showed proteomic changes potentially associated with the autonomic dysfunction observed in ME/CFS patients with POTS. GAP43 was notably upregulated in POTS patient CSF and is involved in neuron growth and development^20^, and its altered expression is associated with neurodegenerative diseases^20,21^. The connection to neuron growth, development, and function was further supported by the differential expression of two other proteins identified in this analysis: VAMP2^22^, and TMEM59L^23^. In contrast, MXRA8 were downregulated in the CSF of patients with POTS. MXRA8 is a receptor for alphaviruses^24^, with implications in the development of musculoskeletal diseases^25^. Altogether, these proteins only meeting nominal significance are likely due to the limited sample size and inherent variability in CSF proteomics^26^.

Synchronous changes within a pathway may reveal biologically important effects even when individual proteins do not reach significance^27^. Therefore, pathway analysis was performed on these data and identified an enrichment of neutrophil degranulation and platelet-related signatures in POTS, and complement cascade and coagulation-related pathways corresponding with severity of ME/CFS. These findings are consistent with previous reports of vascular^28^ and immune^29^ dysregulation in ME/CFS and may reflect inflammatory activation or microvascular instability within the central nervous system. In addition, altered activity in IGFBP-mediated insulin-like growth factor transport suggests potential links to impaired neuroendocrine signaling or metabolic regulation^30^, which have been implicated in ME/CFS pathophysiology. Although these results should be interpreted as exploratory, they provide pathway-level hypotheses grounded in biologically plausible mechanisms that merit further investigation in larger, independent cohorts.

Ratio-based analysis revealed an increase in the YWHAG/NPTX2 ratio by ME/CFS severity, which was shown to be predictive of cognitive impairment in Alzheimer’s disease^18^, suggesting shared features of neuronal adaptation or stress across neuroimmune conditions. This connection to markers of Alzheimer’s disease is further supported by the DEP analysis, as GAP43 has been shown to be elevated in the CSF of Alzheimer’s disease^21^. The ratio-based form of analysis also revealed three additional biologically interpretable ratios of differentially expressed proteins that varied with ME/CFS severity: PDIA4/ABHD14A, CHST15/GALNT16, and C4BPA/NXPH4. These protein ratios span domains of cellular stress, extracellular remodeling, and immune-neuronal interaction.

PDIA4 is involved in oxidative protein folding and endoplasmic reticulum stress responses^31–33^—processes relevant to cellular stress and immune activation observed in ME/CFS. ABHD14A is a predicted cytoplasmic hydrolase with potential roles in neuronal development and metabolic regulation^34,35^, though its function in disease contexts remains understudied. The positive correlation between the PDIA4/ABHD14A ratio and ME/CFS severity therefore reflects dysregulation of cellular stress. CHST15 and GALNT16 are both enzymes involved in glycosylation—CHST15 in sulfation of glycosaminoglycans^36^ and GALNT16 in O-linked glycan initiation^37^. Altered glycan processing may influence extracellular matrix integrity and immune cell signaling^38–40^, both implicated in ME/CFS pathology. Therefore, the positive correlation between this ratio and ME/CFS severity has implications for the role of extracellular remodeling in the pathophysiology of the disease. C4BPA regulates the classical complement pathway^41^, while NXPH4 is a neuronal glycoprotein of the neurexophilin family^42^. Therefore, this ratio may reflect the interplay between immune modulation and neuronal signaling, relevant to the neuroimmune phenotype of ME/CFS. While exploratory, this ratio-based analysis offers distinct mechanistic hypotheses for further investigation in ME/CFS pathophysiology.

In conclusion, these findings from protein-level, ratio-based, and pathway analyses offer mechanistic hypotheses that may inform future biomarker discovery and support the role of CSF proteomics in uncovering central nervous system involvement in ME/CFS. To our knowledge, this is the first mass spectrometry-based proteomics study examining CSF protein differences related to POTS status and disease severity within an ME/CFS cohort, and it provides important evidence for the role of immune dysregulation affecting the central nervous system in ME/CFS pathophysiology, as suggested in previous studies^12–16^. The ratio pairs identified, once validated, will be a valuable contribution to future biomarker discovery, as they may provide a more stable and reproducible measurement.

This study has several limitations. The cohort is limited to participants with ME/CFS, and therefore the analyses cannot determine whether the observed proteomic signals reflect processes specific to POTS or instead represent heterogeneity within ME/CFS or broader dysautonomia- or immune-related biological patterns. The absence of POTS-only and healthy control groups limits the ability to attribute the observed differences uniquely to POTS. In addition, the relatively small subgroup sizes and the lack of protein-level FDR significance in the POTS contrast increase uncertainty and the risk of false-positive findings. Accordingly, pathway analyses derived from nominally associated protein sets should be interpreted as exploratory and hypothesis-generating.

Ultimately, validation in larger, independent cohorts will be essential to confirm these observations and assess their clinical relevance. This would also imply the incorporation of appropriate control groups (POTS without ME/CFS, other orthostatic intolerance phenotypes, healthy controls, and/or disease controls). Larger cohorts would allow for more in-depth sensitivity analyses (e.g. age, sex, BMI, illness duration, medications) and effect size calculations alongside confidence intervals and multiple-testing controls. Orthogonal validation (e.g., targeted MS/ELISA) for leading candidates would further strengthen the findings.

## Methods

### Ethics declarations

Ethical approval was granted by the Swedish Ethical Review Authority (#2019–03510 and #2022–06786-02), and all participants provided written informed consent. All methods were performed in accordance with the relevant guidelines and regulations.

### Sex as a biological variable

This study included male and female participants. The sample size was not sufficient for evaluating sex differences in protein expression or subsequent pathway and ratio-based analyses.

### Study participants and clinical assessment

CSF samples initially were obtained from 34 individuals diagnosed with ME/CFS according to the Canadian Consensus Criteria (CCC). Healthy control CSF samples were not collected due to ethical constraints restricting lumbar puncture to symptomatic individuals. Each participant underwent comprehensive clinical evaluation, including assessments of dysautonomia (e.g., POTS), neurological status, demographic background, disease history, and patient-reported outcomes using EQ-5D and RAND-36 instruments. General joint hypermobility was determined by using the Beighton score and the 5PQ questionnaire, as described in Malfait, et al.^43^. A tilt table test was performed to determine POTS— defined as a sustained increase in heart rate of ≥ 30 beats per minute within 10 min of tilting on an electronic tilt table, accompanied by characteristic orthostatic symptoms in the absence of postural hypotension. Prior to tilting, at least three consecutive supine blood pressure and heart rate measurements were taken to confirm hemodynamic stability. This abbreviated procedure is consistent with the diagnostic criteria outlined by Freeman et al.^44^, and was selected to allow standardized yet clinically feasible assessment in a non-cardiology outpatient setting.

A categorical disease severity score (mild, moderate, severe) was assigned to each participant through clinical consensus, supported by symptom profiles.

CSF was collected under aseptic conditions by lumbar puncture with local anesthesia, using sterile polypropylene tubes placed on ice. Samples were immediately centrifuged (2000 × g, 15 min, 4 °C), and supernatants aliquoted into 200 μL cryotubes and stored at − 80 °C. No samples were previously thawed. Three participants were excluded due to blood contamination (n = 2) or abnormal metabolic parameters (n = 1), resulting in a final dataset of 31 patients.

### Mass spectrometry-based proteomics

CSF proteomic profiling was conducted using a QExactive Plus Orbitrap mass spectrometer (Thermo Fisher Scientific, Bremen, Germany) coupled to an EASY-nLC 1000 liquid chromatography system. Peptides were separated using a C18 pre-column (2 cm, 100 μm ID) and analytical column (10 cm, 75 μm ID). A 150-min linear gradient elution from 4 to 100% acetonitrile was applied at 250 nL/min. Mass spectra were acquired in positive ion mode over the m/z range of 400–1750 with a resolution of 70,000. The top 10 ions were subjected to higher-energy collisional dissociation (HCD) at 25% normalized energy, with fragment spectra collected at 17,500 resolution.

### Protein identification and quantification

Raw spectra were processed using MaxQuant (v2.2.0.0)^45^ and the Andromeda search engine against the UniProt human FASTA database (June 2022)^46^. Search parameters included a 4.5 ppm tolerance for precursor ions and 0.5 Da for MS/MS fragments. Trypsin was specified as the digestion enzyme, permitting up to two missed cleavages. Carbamidomethylation of cysteine was set as a fixed modification, while methionine oxidation and N-terminal acetylation were set as variable modifications. Protein identifications were filtered at a 1% FDR.

Label-free quantification (LFQ) was performed using default MaxQuant settings, excluding intensity-based absolute quantification. LFQ values were log₂-transformed, and proteins detected in at least eleven (33.3%) samples were retained for downstream analyses. Abundance values were calculated from the mean intensity of the three most abundant peptides per protein.

### Principal component analysis and differential expression

To explore proteomic variance, a principal component analysis was conducted on log₂-transformed LFQ data using the prcomp function in R. Visualization was performed with ggplot2^47^. Protein intensities were normalized using variance stabilizing normalization (VSN)^48^ via the DEP::normalize_vsn()^49^ function. Differential expression analysis across POTS status and categorical severity groups was performed using the limma package (v3.14)^50^, with empirical Bayes moderation and quantile normalization. The design matrix was constructed to include both POTS status (POTS vs. NoPOTS) and Disease Severity (Mild, Moderate, Severe) as covariates. Model fitting was followed by empirical Bayes moderation. Detailed results of this analysis, including fold changes and adjusted p-values, are provided in Supplementary Table 1.

### Pathway enrichment analysis

To investigate biological pathways associated with ME/CFS severity and POTS status, pathway enrichment was performed using IPA (QIAGEN)^51^. The input set consisted of differentially expressed proteins filtered based on nominal thresholds (p-value < 0.05 and fold change > 1.5), representing exploratory candidates for downstream analysis. Canonical pathway analysis was conducted using IPA’s default settings.

### Pairwise protein ratio analysis across ME/CFS severity

To explore coordinated protein expression changes associated with ME/CFS severity, a systematic analysis of pairwise protein abundance ratios derived from DEPs was conducted. DEPs were pre-filtered based on nominal statistical criteria (p < 0.05 and fold change > 1.5) from the severity-based differential expression analysis. All unique pairwise combinations of these DEPs were generated, and the abundance ratio for each protein pair was calculated per participant. CSF samples were grouped into three clinician-defined ME/CFS severity categories (mild, moderate, severe), which were encoded as an ordinal numeric variable (1 = mild, 2 = moderate, 3 = severe) to facilitate trend analysis.

### Statistics

Multiple testing correction was applied to the DEP analysis using the Benjamini–Hochberg procedure^52^ to control the FDR. As no proteins met the stringent significance threshold (adjusted p-value < 0.05 and absolute fold change > 1.5), results were reported based on nominal criteria: proteins with an absolute fold change > 1.5 and nominal p-value < 0.05 were considered nominally significant.

Pathway significance was assessed using IPA’s right-tailed Fisher’s exact test, with Benjamini–Hochberg adjusted p-values < 0.05 used to define statistically enriched pathways. IPA’s activation z-score algorithm was applied to infer pathway activation or inhibition based on the direction of protein-level changes.

For each ratio, its relationship with disease severity was assessed using two complementary nonparametric methods:

- Spearman correlation between the ratio and severity score, with 95% confidence intervals estimated via bootstrap resampling (1000 iterations);
- Kruskal–Wallis tests to assess overall group-wise differences in ratio distributions without assuming normality or equal variances.

To control for multiple hypothesis testing across all ratio comparisons, q-values were calculated using R::qvalue package^53^. Only protein ratios with q < 0.05 and absolute Spearman correlation ≥ 0.75 were considered statistically meaningful and retained for downstream interpretation.

## Supplementary Information

Supplementary Information.

## Data Availability

The data underlying this article are provided in the Supporting Data Values file, including the normalized protein quantification profiles obtained using VSN, and the results of DEP analyses performed using the limma package. Specifically, DEPs comparing POTS to non-POTS groups, and DEPs across different categorical severity levels, are included in Supplementary Table 1. The clinical-level raw data cannot be publicly deposited due to IRB/GDPR constraints, but aggregated summaries and processed matrices can be shared upon reasonable request to the authors. The mass spectrometry proteomics data have been deposited to the ProteomeXchange Consortium via the PRIDE partner repository with the dataset identifier PXD076216^54, 55^.
